# Post mortem cadaveric and imaging mapping analysis of the influence of cochlear implants on cMRI assessment regarding implant positioning and artifact formation

**DOI:** 10.1007/s00405-024-09164-0

**Published:** 2024-12-30

**Authors:** P. Arnold, L. Fries, R. L. Beck, S. Granitzer, M. Reich, A. Aschendorff, S. Arndt, M. C. Ketterer

**Affiliations:** 1 Department of Otorhinolaryngology – Head and Neck Surgery, Medical Center, Faculty of Medicine, University of Freiburg, Killianstrasse 5, 79106 Freiburg, Germany; 2 Oticon Medical, 2720 Chemin Saint-Bernard, 06220 Vallauris, France; 3 Faculty of Medicine, Eye Center, Albert-Ludwigs University Freiburg, 79085 Freiburg, Germany; 4 Department of Radiology, Medical Center, Faculty of Medicine, University of Freiburg, Freiburg, Germany

**Keywords:** MRI, Cochlear implant, Artifact, Magnet, Cranial imaging

## Abstract

**Objectives:**

In times of an aging society and considering the escalating health economic costs, the indications for imaging, particularly magnetic resonance imaging (MRI), must be carefully considered and strictly adhered to. This cadaver study aims to examine the influence of cochlear implant (CI) on the assessment of intracranial structures, artifact formation, and size in cranial MRI (cMRI). Furthermore, it seeks to evaluate the potential limitations in the interpretability and diagnostic value of cMRI in CI patients. Additionally, the study investigates the imaging of the brain stem and the internal ear canal and the feasibility of excluding cholesteatomas in cMRI for CI patients.

**Materials and methods:**

Two cadaveric specimens were implanted with cochlear implants at varying angular positions (90°, 120°, and 135°), both unilaterally and bilaterally, with and without magnet in situ. MRI acquisition consisted of sequences commonly used in brain MRI scans (T_1_-MP-RAGE, T_2_-TSE, T_1_-TIRM, DWI, CISS). Subsequently, the obtained MRI images were manually juxtaposed with a reference brain from the Computational Anatomy Toolbox CAT12. The size and formation of artifacts were scrutinized to ascertain the assessability of 22 predefined intracranial structures. Furthermore, the internal auditory canal, middle ear and mastoid were evaluated.

**Results:**

The cadaveric head mapping facilitated the analysis of all 22 predefined intracranial structures. Artifacts were assessed in terms of their minimum and maximum impact on image comparability. Image quality and assessability were stratified into four categories (0–25%, 25–50%, 50–75%, and 75–100% of assessability restriction). The visualization of the central, temporal, parietal, and frontal lobes was contingent upon CI positioning and the choice of imaging sequence. Diffusion-weighted cMRI proved inadequate for monitoring cholesteatoma recurrence in ipsilateral CI patients, regardless of magnet presence. The ipsilateral internal auditory canal was inadequately visualized in both magnet-present and magnet-absent conditions. We divided our results into four categories. Category 3 (orange) indicates considerable limitations, while category 4 (red) indicates no interpretability, as the image is entirely obscured by artifacts.

**Conclusion:**

This study provides detailed predictive power for the assessability and therefore the relevance of performing cMRIs in CI patients. We advocate consulting the relevant CI center if artifact overlay exceeds 50% (categories 3 and 4), to evaluate magnet explantation and reassess the necessity of cMRI. When suspecting cholesteatoma or cholesteatoma recurrences in patients with ipsilateral cochlear implants, diagnostic investigation should preferably be pursued surgically, as the necessary MRI sequences are prone to artifact interference, even in the absence of a magnet. The ipsilateral internal auditory canal remains inadequately evaluable with a magnet in situ, while without the magnet, only rudimentary assessments can be made across most sequences.

## Introduction

In the past, magnetic resonance imaging (MRI) has posed either a contraindication for cochlear implant (CI) users or an increased risk for implant magnet dislocation, magnet weakening, pain, or implant defect, necessitating reimplantation [3, 8, 13, 20, 21]. Reimplantation is correlated with possibly diminished postoperative speech perception and cochlear trauma, leading to subsequent fibrosis [2, 10, 12]. Initially, in 1995, MRI was authorized for CI users but only at a low dose of 0.2 Tesla MRI [7]. Consequently, manufacturers developed freely rotating magnets, first established by MED-EL, to mitigate the risk of magnet dislocation [3]. Nevertheless, with the increasing indication of CI surgery not only in single-sided deafness (SSD) and asymmetric hearing loss (AHL) patients [1, 11] but also in elderly patients [14, 15], the need for adequate MRI assessment, e.g., to exclude or evaluate a stroke, is growing. Furthermore, patients with bilateral profound hearing loss are increasingly undergoing bilateral implantation [17]. The prevalence of cMRI in CI patients is significantly higher, around 49% (Royal College of Radiologists [23]). Cranial MRI scans constitute approximately 20 to 30% of all performed MRIs. The assessability of intracranial lobes, the brainstem, and the inner ear canal depends on the interaction of the CI magnet and the magnetic resonance field. Schreyer et al. [18] performed a cost analysis of contrast-enhanced cranial MRI and could demonstrate that MRI indication has to be clear and substantive to reduce unnecessary economic burdens on healthcare systems.

Previous studies [3, 4, 24] have described the influence of CI positioning on MRI artifacts in Ultra 3D CIs of Advanced Bionics and Cochlear^™^ 512 magnets. Todt et al. [24] first described that specific MRI sequences can reduce the size of the CI artifact in MRI scans. Canzi et al. [4] conducted a preclinical study by wrapping the CI on a model and described different artifact involvements depending on the angular CI position. However, the CI was not implanted but wrapped around the head, potentially introducing bias.

The aim of this study is to evaluate CI positioning, artifact formation, and the size of Oticon medical CIs under 3 Tesla cranial MRI. Furthermore, we aim to examine the influence of CI position on the assessment of intracranial structures, artifact formation, and size to predict the possibility of representation before conducting an MRI, thereby reducing medically unnecessary MRI examinations with magnets, both medically and economically. The internal auditory canal and the brainstem were regarded as one of the main regions of interest. Additionally, we aimed to investigate the possibility of excluding cholesteatomas of the middle ear or mastoid in CI patients using cranial MRI.

## Methods

### Surgical procedure

**Fig. 1:**
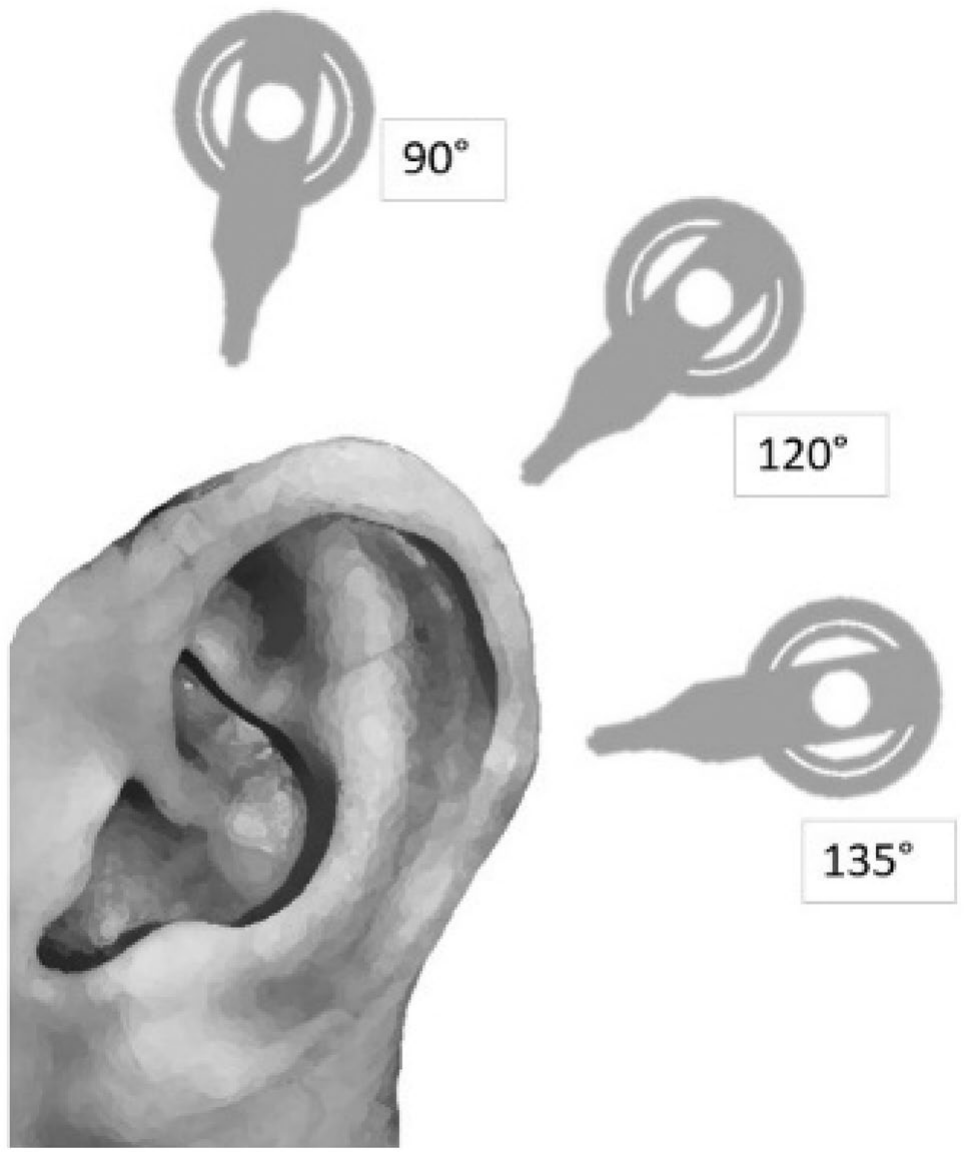
Illustration of the three different evaluated implant positions in 90°, 120° and 135° for both cadaveric heads

This study was conducted on two cadaver heads, which were implanted in different angular positions (90°, 120°, and 135°), both uni- and bilaterally, with and without a magnet in situ, as schematically illustrated in Fig. 1. The line from the nasion to the outer ear canal was used to define the various examined angles, as established by Canzi et al. [4]. We did not conduct the measurements in the three different angular positions to compare the positions with each other, but rather to cover the range of widths of the already implanted cochlear implant patients, as the angle is chosen by the surgeon in the range of 90° to 135°, but mostly at 120°. All unilateral CIs were implanted on the left side. Prior to the study, both heads were inspected for malformations and dysmorphisms and underwent CT morphological examination. Particularly, no malformations and/or abnormalities of the petrous bones were apparent in both cases. The study received approval from the Ethics Committee of the University Hospital of Albert-Ludwigs University Freiburg. Both cadaveric heads were body donors within the scope of a skull base course and volunteered to donate their bodies for scientific and educational purposes during their lifetimes (Ethics Committee Number: 24-1178-S1). This study is registered (DRKS—number: 00034859; FRKS—number 005200). Oticon Medical provided a total of 4 implants for the implementation of this study. These implants were implanted at different angles with and without magnets into the cadaver heads and fixed to the cortical bone using screws. The retroauricular access was closed with sutures. There was no wrapping or bandaging of the cadaver heads. The sequence of the performed MRI examinations is listed in Table 1 (study protocol). MRI numbers 1 to 9 designate the respective MRI conditions for head 1 or head 2 and should consistently be used as labels for the results presented in Tables 3 and 4. Some MRI sections were performed on both heads, particularly the unilateral measurements with the magnet in situ and the 135° angle (most commonly used in our department) without the magnet in situ, to demonstrate the validity and assessability of the measurements.

**Table 1:** Study protocol of both cadaveric heads, implanted in different angular positions, with and without magnet (unilateral = left side implanted)

MRI number	Head 1	Head 2
1	Bilateral with magnet (135°)	
2	Bilateral without magnet (135°)	
3		Unilateral with magnet (135°)
4	Unilateral without magnet (135°)	
5	Unilateral with magnet (120°)	
6		Unilateral without magnet (135°)
7	Unilateral with magnet (90°)	
8		Unilateral with magnet (120°)
9		Unilateral with magnet (90°)

### Imaging analysis

MRI scans were performed with a 3 T scanner (MAGNETOM Prisma, Siemens Healthcare) with a 64-channel head and neck coil. MRI sequences consisted of sagittal 3D Magnetization Prepared—RApid Gradient Echo (MP-RAGE) Table 2 gives technical information, voxel size, TR, TE and acquisition time of the examined sequences. MRI acquisition was conducted across varying implant placements, as outlined in Table 1. Manual alignment was subsequently conducted utilizing our in-house imaging and postprocessing platform, NORA (www.nora-imaging.com), to align the cadaveric skull as visualized in the MRI images with a standard brain template from the Computational Anatomy Toolbox for the analysis of structural MRI data (CAT12). CAT12, an extension of the SPM12 software, is widely used for structural brain MRI analysis, offering morphometric tools that integrate voxel-based, surface-based, and region-based approaches. The CAT tool is frequently described in studies as an outstanding and innovative tool for human brain mapping, featuring multiple quality controls (Gaser et al. [39]). Several studies have validated the CAT12 tool's labeling by comparing it to manual labeling (Gaser et al. [39]). As outlined by Gaser et al. [39], the use of CAT12 has been proven to be accurate, sensitive, reliable, and reproducible. It has been compared to manual anatomy as well as other software, consistently demonstrating improvements and outperforming other commonly used neuroimaging tools (Gaser et al. [39]), [26–37].

**Table 2:** Examined sequences with voxel size, TR, TE and acquisition time

Sequences	Voxel size	TR	TE	Acquisition time
T_1_-weighted images	1.0 × 1.0 × 1.0 mm	2300 ms	2.26 ms	234 s
T_2_-weighted turbo spin echo (TSE) images	0.4 × 0.4 × 5 mm	6440 ms	110 ms	169
T_1_-weighted turbo inversion recovery magnitude (TIRM) images	0.7 × 0.7 × 5 mm	2000 ms	9 ms	174 s
T_2_-weighted constructive interference in steady state (CISS) images	0.5 × 0.5 × 0.5 mm	8.39 ms	3.91 ms	403 s
Diffusion-weighted images	0.6 × 0.6 × 5 mm	3500 ms	85 ms	47 s

**Fig. 2:**
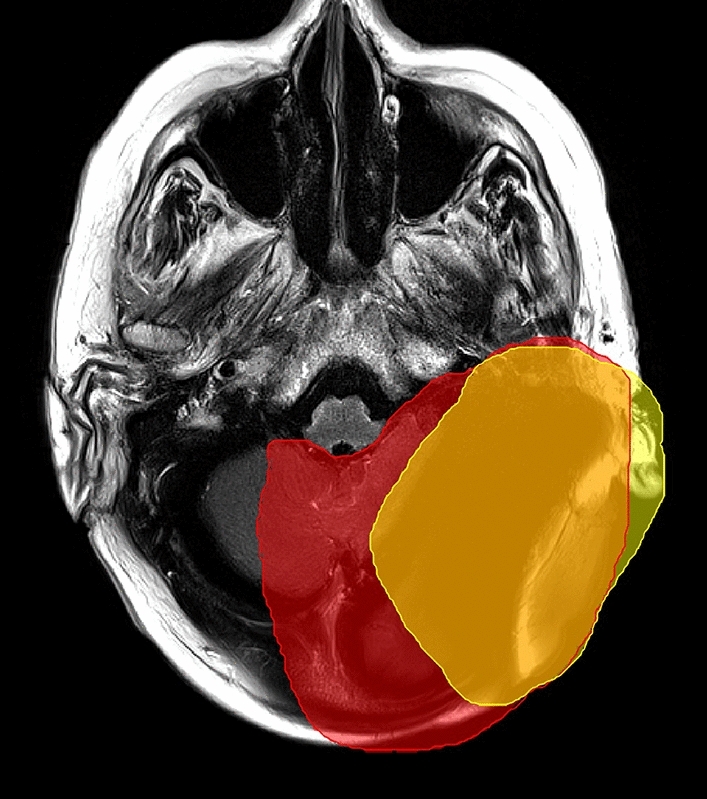
Unilateral 135° CI position with magnet in situ demonstrating the definition of minimal (yellow) and maximal (red) artifact. We defined the cutoff between minimal and maximal artifact regions as follows: the minimal artifact region does not allow for assessment of imaging interpretability, whereas the maximal artifact region may still depict large tumors, hemorrhages, or malformations larger than 1 cm

The formation and dimensions of artifacts were scrutinized to evaluate the visibility of intracranial structures. These artifacts were manually segmented in NORA by two head and neck surgeons with extensive expertise in neuroradiology in combination with a radiologist with two years experience in neuroradiology and three years of experience in otorhinolaryngology, who also studied physics. We defined two artifact regions: a minimal artefact region, which exhibits strong susceptibility artefacts, impeding radiological evaluation, and a maximal artifact region, which extends to the unperturbed and artifact free image area (see Fig. 2). The cutoff between minimal and maximal artifact region was that the minimum artefact region provides no assessment of imaging interpretability, whereas with maximum artefact region may still depict large tumors, bleedings, or malformations > 1 cm.

A total of 22 discernible intracranial structures were identified, as detailed in Table 3. Furthermore, the internal auditory canal was evaluated as described in Table 4 and the middle ear and mastoid were evaluated. The volume overlap of the segmented artifacts with the selected areas of the WFU_PickAtlas framework was calculated [9] (https://www.nitrc.org/projects/wfu_pickatlas), previously described by Takamura et al. [22] and Maldjian et al. [16]. The interpretability of segmented and analyzed MRI images was categorized into four categories (0–25%, 25–50%, 50–75%, and 75–100% assessability restriction).

**Table 3:**
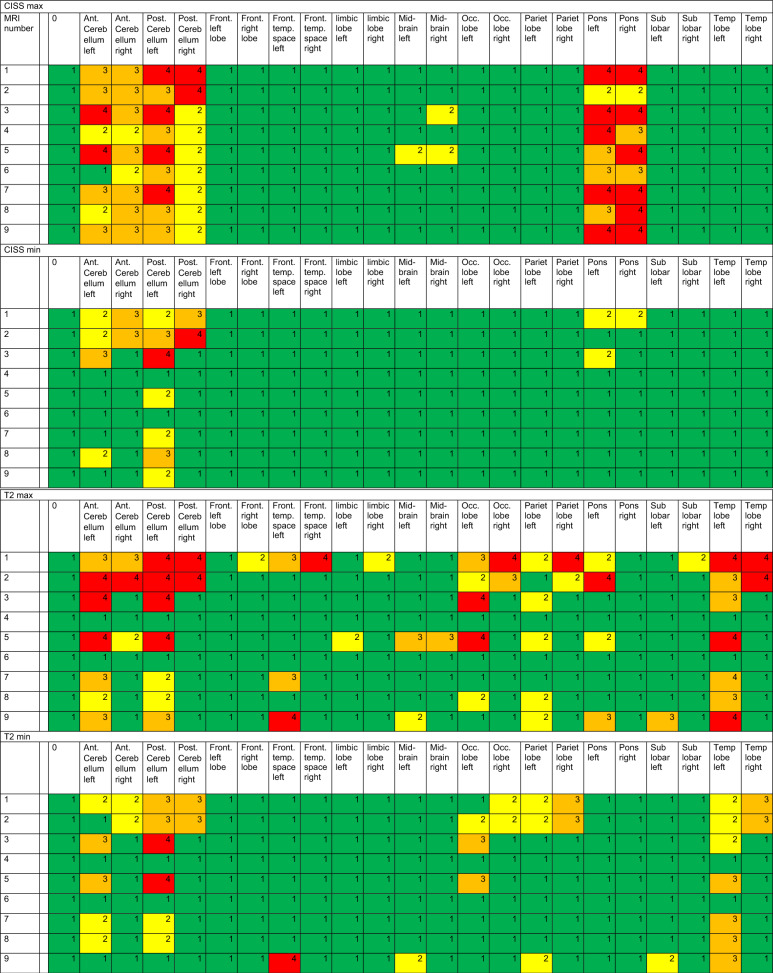

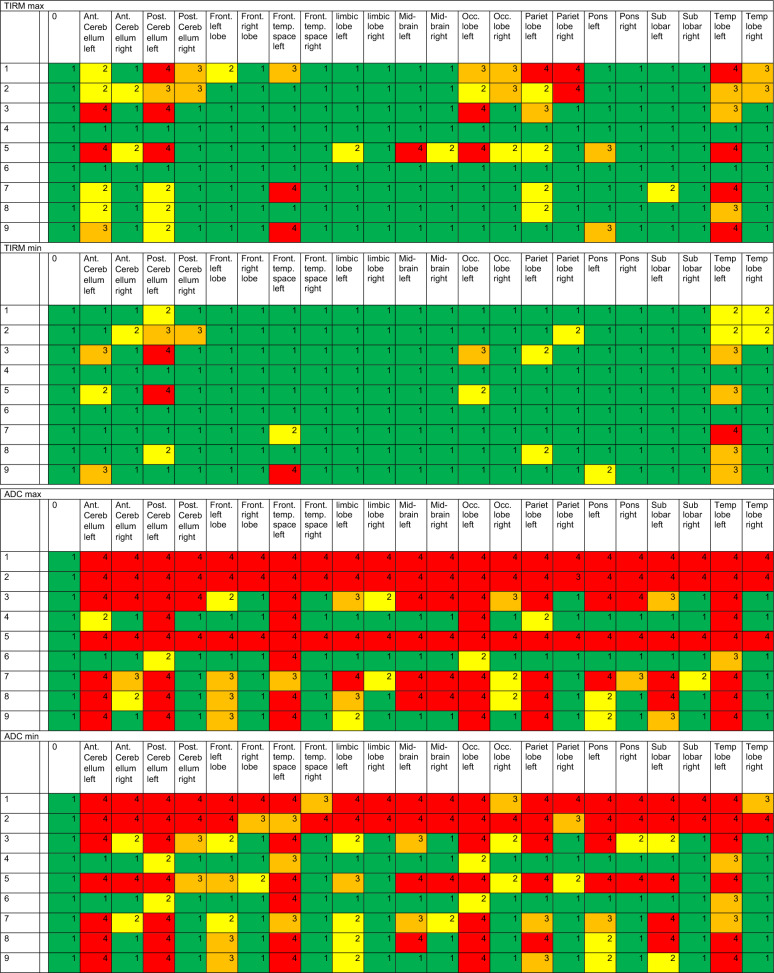

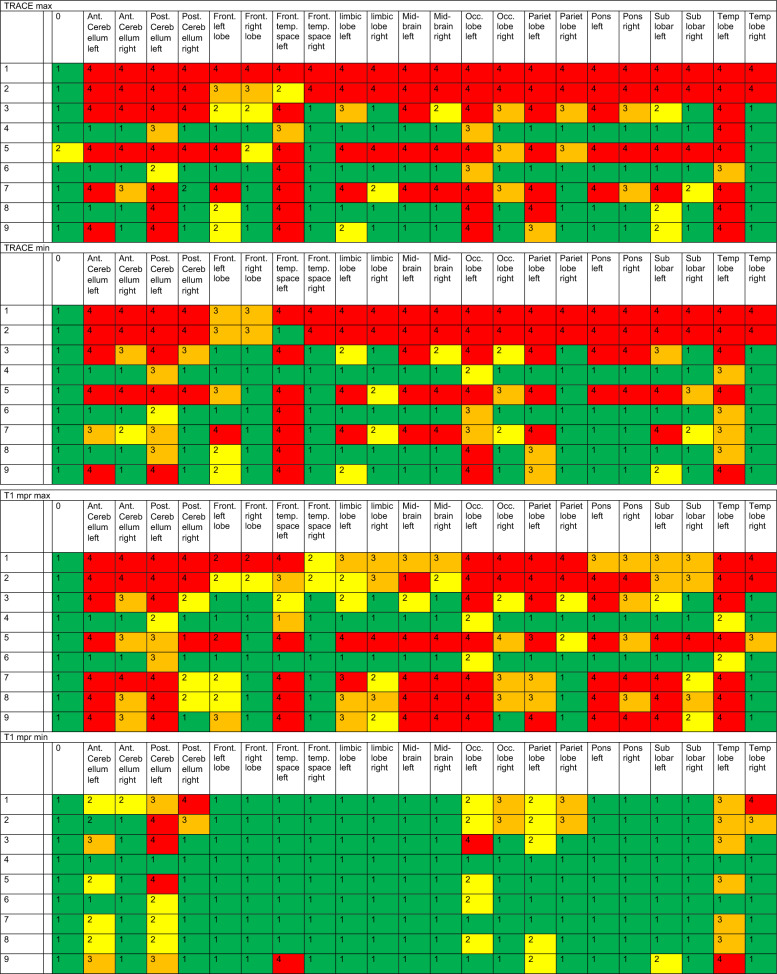
A total of 22 discernible intracranial structures were identified and examined in different sequences

Min and Max is demonstrating the minimum (min) and maximum (max) artifact condition in the sequences CISS, T2, TIRM, ADC, TRACE, and T1mpr. Clear significance and absence of limitations due to artifacts are anticipated at category 1 (green), while category 2 (yellow) denotes initial limitations, particularly at maximum artifact intensity. Category 3 (orange) implies considerable limitations, whereas category 4 (red) implies no interpretability as the image is entirely obscured by artifacts. On the vertical axis, the MRI conditions for head 1 or head 2 are listed, as described in detail in Table 1. All unilateral CIs were implanted on the left side

**Table 4:**
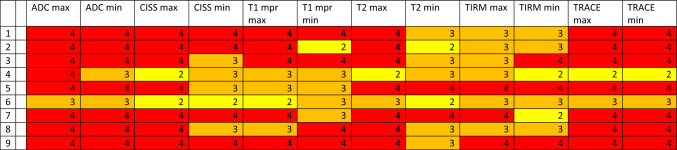
Minimal (min) and maximal (max) artifact condition in the sequences CISS, T2, TIRM, ADC, TRACE, and T1mpr for the internal ear canal

Clear significance and absence of limitations due to artifacts are anticipated at category 1 (green), while category 2 (yellow) denotes initial limitations, particularly at maximum artifact intensity. Category 3 (orange) implies considerable limitations, whereas category 4 (red) implies no interpretability as the image is entirely obscured by artifacts. On the vertical axis, the MRI conditions for head 1 or head 2 are listed, as described in detail in Table 1

## Results

Table 3 illustrates the significance and utility of conducting cMRI utilizing CISS, T2, TIRM, ADC, TRACE, and T1mpr sequences. We defined four categories referring to a division of image qualities that share common characteristics and interpretability. Clear significance and the absence of limitations due to artifacts are anticipated in category 1 (green) with visibility restrictions of less than 25%. Category 2 (yellow) indicates initial limitations, but still better than 50% of impairments, particularly at maximum artifact intensity. Category 3 (orange) suggests considerable limitations, up to 75%, while category 4 (red) indicates no interpretability, as the image is entirely obscured by artifacts in over 75% of cases. Table 3 clearly shows the limitations of MRI imaging depending on the angle of the implant, but especially the difference between with and without the implant magnet. Intracranial imaging in bilateral CI recipients with the magnet (MRI number 1) is difficult to assess, particularly in ADC, TRACE, and T1mpr sequences. However, these sequences are also challenging to evaluate for many anatomical regions in bilateral CI without the magnet (see Table 3, MRI number 2).

**Fig. 3:**
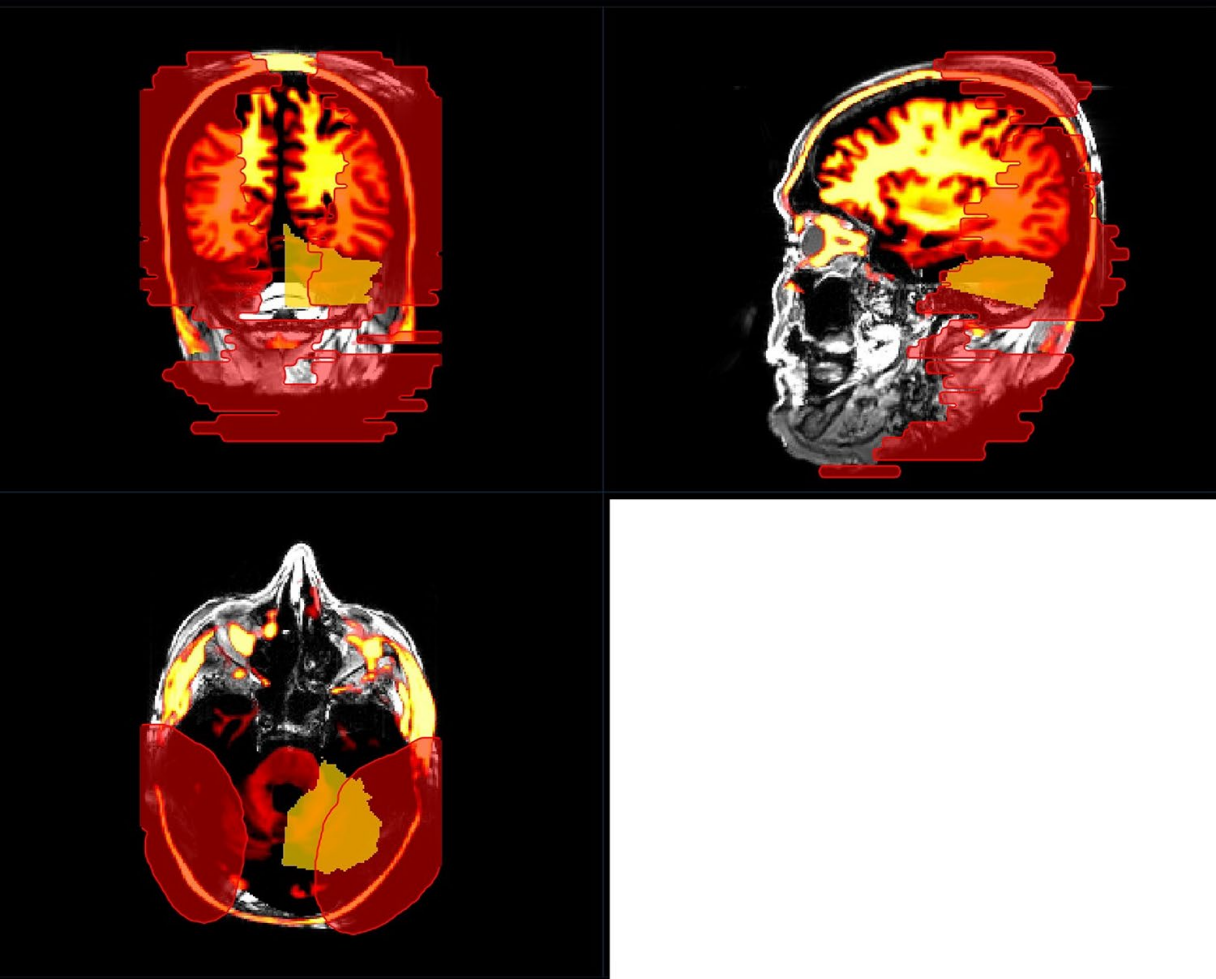
Mapping imaging analysis of cadaveric heads, with modeled intracranial structures. In red the maximal artifact regions, that cannot be interpreted, here for the examined region of the left cerebellum (demonstrated in orange) (condition of CI = bilateral, 135° with magnet in situ) (MRI, sequence: CISS, in three dimensional reconstruction)

**Fig. 4:**
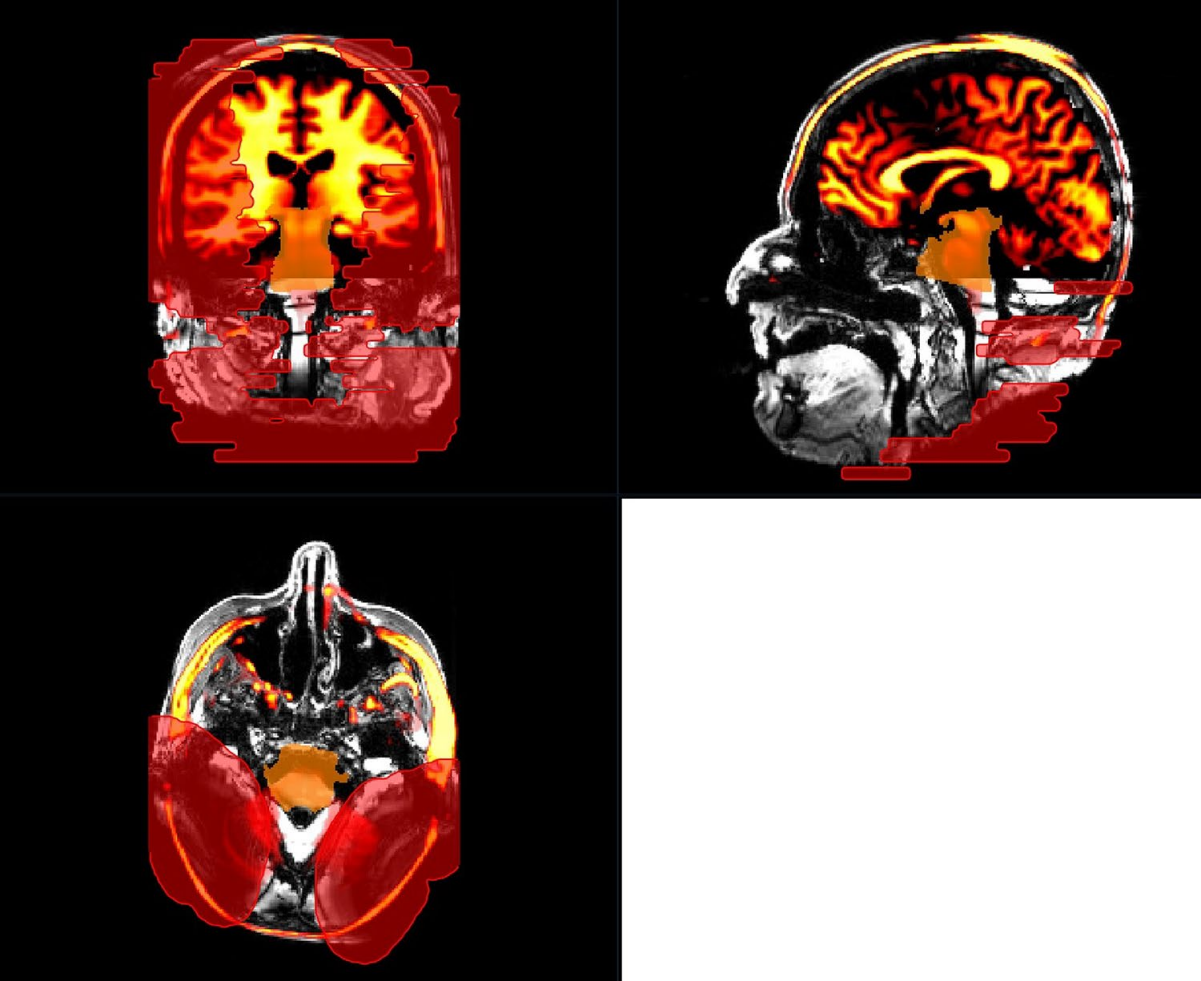
Mapping imaging analysis of cadaveric heads, with modeled intracranial structures. In red the maximal artifact regions, that can not be interpreted, here for the examined region of the brain stem (condition of CI = bilateral, 135° with magnet in situ) (MRI, sequence: CISS, in three dimensional reconstruction)

An illustration of the minimum (min) and maximum (max) artefact regions is depicted in Fig. 2. The minimum artefact region (depicted in yellow) allows no assessment of the MRI whatsoever, whereas the maximum artefact region (depicted in red), allows a restricted evaluation of the brain tissue and surrounding structures. For example, Fig. 3 presents Cadaver Head 1 with bilateral implants and the magnet in situ positioned at 135° on both sides, with color-mapped intracranial structures (yellow: left cerebellum). Here, maximal artefact region is denoted in red segmentation, focusing on the left cerebellum, which, as shown in Table 3, is overlaid with artifacts by over 50%. Figure 4 similarly displays Cadaver Head 1 with bilateral implants and the magnet in situ positioned at 135° on both sides, featuring color-mapped intracranial structures (orange: brainstem). Again, the maximal artefact region is indicated in red segmentation, focusing on the brainstem, which, as shown in Table 3, is overlaid with artifacts by over 50%.

Regarding Diffusion-weighted sequences (Table 3, ADC and TRACE sequences) visuability is completely artefacted (category 4, red) for CI condition bilateral with and without magnet. The region of the temporal bone and mastoid (for MRI exclusion of cholesteatoma in the middle ear and mastoid) was not feasible in both cadaveric heads due to artifacts with ipsilateral CI condition with magnet in situ. It was found that an ipsilateral cochlear implant with an in situ magnet provides no diagnostic value for assessing either the ipsilateral or contralateral side in diffusion-weighted imaging (ADC, TRACE), and therefore no conclusions can be made regarding the exclusion of a cholesteatoma in the middle ear or mastoid. An ipsilateral cochlear implant without the magnet might allow for the depiction of a contralateral cholesteatoma, but not the ipsilateral one.

As depicted in Table 4, imaging and interpretability in CISS and T1 for the internal auditory are only moderately feasible in the condition of CI 135° without the magnet in situ and only with ipsilateral implant. Nevertheless, both imaging conditions of cadaver 1 and 2 with unilateral without magnet in situ CI with 135° insertion angle demonstrate that this interpretability is not highly effective and still leads to a moderate restriction in interpretability between 25 and 50% (category 2).

## Discussion

### Study design, background and MRI artifacts

This study aimed to furnish radiologists and neuroradiologists with the capacity to adequately evaluate the appropriateness prior to conducting cMRI in CI patients. MRI remains the most economically burdensome cross-sectional imaging modality available and hence necessitates cautious and judicious indication. Alongside interactional issues such as implant displacement, pain, device failure, magnet displacement, demagnetization, and potential skin and implant bed irritation, the presence of artifacts poses a significant challenge in MRI diagnostics for CI patients. Although MED-EL initially introduced a novel technique utilizing a rotating magnet to alleviate implant-related issues, caused by torque effects, which is important for the patient and facilitated MRI examinations in other regions of the body, artifacts in cMRI persist as a challenge. We could comprehensively demonstrate the extent to which diagnostic reliability in cMRI is feasible for different inquiries and whether the execution of an MRI is advisable at all. Overall, few studies have been conducted on the visualization and assessability of intracranial structures in MRI for CI patients, and our work represents a significant contribution in this area. One main research group [3, 4] has evaluated this impact so far. Multiple studies [3, 5, 6, 19] have suggested that improved MRI image quality can be achieved with planar and spin echo sequences. Artifact sizes have been reported, ranging from a 55 mm radius [6] to 49.6 to 56.7 mm [3] in axial planar sequences, corroborated by Sharon et al. [19] in one of the largest study cohorts to date. Wagner et al. [25] confirmed these findings with a 50 mm artifact radius in cadaveric heads for both 1.6 and 3 T MRI scans. Nevertheless, Canzi et al. [3] evaluated their results based on the assessment of one plane and described that in the coronal plane, 9 out of 14 regions were assessable. In contrast, we evaluated our results using three-dimensional reconstruction, which significantly enhanced their validity in clinical application.

### MRI analysis and interpretation

As the data from this study for the evaluation of cMRI demonstrate (see Table 3), interpretability, particularly ipsilateral to the CI, is influenced by the target anatomical region, the position of the implant, and, most notably, the presence of the CI magnet. To the best of our knowledge, this is one of the first studies to examine this effect both ipsilateral and contralateral to a CI, as well as in cases of bilateral CIs. Canzi et al. [3] assessed 14 different intracranial structures, categorizing them into three groups: high-quality imaging (HQ), images with artifacts but still diagnostic (A), and non-assessable images (NA). They reported that 9 out of 14 regions were either HQ or A for interpretation in cases of ipsilateral CI with the magnet in situ. While our results differ somewhat from theirs, comparing our 22 regions to their 14, we find considerable similarities when looking at specific anatomical areas. For example, we confirm their findings [3] for the occipital lobe, which was classified as NA for contralateral and bilateral situations and artifact-affected (A) in the ipsilateral CI condition. We can further confirm the significantly limited visibility of the occipital lobe, especially in T1, T2, and TIRM sequences, under ipsilateral, contralateral, and bilateral conditions (see Table 3).

Regarding bilateral CI conditions, our results largely align with those of [3], who suggested a mutual interaction between the two magnets, leading to additional artifacts. In all 14 anatomical regions they assessed, they described either non-assessability or artifact involvement. In addition to previous studies, however, we examined a key criterion: the condition of the CI after explantation of the CI magnet. Our findings show that, in bilateral CI cases post-magnet explantation, MRI visibility significantly improves, though it remains limited, especially in diffusion-weighted sequences, which are still barely assessable.

Canzi et al. [3] compared unilateral and bilateral CIs for ipsilateral and contralateral intracranial structures using the Ultra 3D CI (Slim J) of Advanced Bionics, with the magnet positioned at a 135° angle. However, it should be noted that their studies were conducted on cadaver heads, and the MRI assessments were based on brains preserved in a formaldehyde-water solution. Therefore, their results are not directly comparable to our in vivo measurements, as they did not use a brain mapping system like we did. In their follow-up study [4], they compared four different magnet angles and concluded that an angle of 160° might provide better visualization of the frontal lobes. However, this configuration is neither aesthetically nor audiologically feasible and is incompatible with patient comfort. The primary objective of our study was not to directly compare the various angle positions, but rather to predict as precisely as possible the implications for each already implanted CI patient. Nevertheless, we only examined the implant of one manufacturer, and therefore conclusions regarding other CI brands should be drawn with caution. As patients are typically implanted at angles between 90 and 135°, this study can now provide some guidance to clinicians considering cMRI for CI patients. Table 3 illustrates to what extent cMRI in the required sequences is meaningful or not depending on the angle of the implant and whether the patient is unilaterally or bilaterally implanted. In contrast to previous studies [3, 4], we conducted our investigations not only using a 1.5 Tesla machine but also a 3 Tesla machine, which is much closer to today's clinical routine. However, this also explains the somewhat poorer visibility results in our study.

### Diffusion-weighted imaging for cholesteatoma evaluation

Diffusion-weighted imaging is a useful technique for the evaluation of cholesteatomas [38]. It can be used to detect them when the physical examination is difficult and computed tomography findings are equivocal, and it is especially useful in the evaluation of recurrent cholesteatoma. Initial Diffusion-weighted imaging techniques only detected larger cholesteatomas, > 5 mm, due to limitations of section thickness and prominent skull base artifacts. Newer techniques allow detection of smaller lesions and may be sufficient to replace second-look surgery in patients with prior cholesteatoma resection. For instance, diffusion sequences in this study revealed that a definitive exclusion of cholesteatoma in CI patients in the middle ear of the mastoid is scarcely achievable with MRI alone, necessitating a surgical exclusion via tympanoscopy in cases of initial suspicion or a second-look operation in cases of recurrence. These findings significantly enhance the conclusions of previous studies [3, 4] by providing substantial insights into the evaluation potential of cholesteatomas in CI patients.

### Evaluation of the internal auditory canal

In our study, as shown in Table 4, the evaluability of the internal auditory canal in CI patients was found to be barely feasible ipsilaterally with the magnet in situ, and only slightly improved without the magnet. The consideration of a contraindicated cMRI remains necessary, particularly in follow-up cases post-vestibular schwannoma. However, Canzi et al. [3] reported 'high-quality' imaging of the contralateral internal auditory canal and artifact-affected, yet diagnostically assessable, imaging for both bilateral and ipsilateral conditions. Possible reasons for this discrepancy may include the absence of three-dimensional reconstruction in their study, the use of only a 1.5 Tesla MRI device, and the addition of our measurements without the magnet, which likely provided further valuable insights. Further investigations comparing the respective differences across implants should be conducted.

## Conclusion

To the best of our knowledge, this is the first study employing software-based analysis and double-checked control by radiology and head and neck surgery experts evaluating this topic of high research interest and increasing importance. Nonetheless, we must acknowledge the limitation of this study in that we did not compare different implants from different manufacturers. In conclusion, this study provides detailed predictive power for the assessability and, therefore, the relevance of performing cMRIs in CI patients. We advocate consulting the relevant CI center if artifact overlay exceeds 50% (categories 3 and 4), to deliberate magnet explantation and reassess the necessity of MRI. Cholesteatoma recurrences in CI patients may preferably be surgically addressed, as the requisite MRI sequences are inherently susceptible to artifact interference, even in the absence of a magnet. Therefore, a critical discussion about second-look surgery following past cholesteatoma in CI patients is necessary due to the lack of reliable MRI imaging capability. The ipsilateral internal auditory canal remains inadequately evaluable with a magnet in situ, while without the magnet, only rudimentary assessments can be made across most sequences.

## Data Availability

The data that support the findings of this study are available on request from the corresponding author. The data are not publicly available due to ethical reasons.
